# Danggui Shaoyao San Alleviates Early Cognitive Impairment in Alzheimer's Disease Mice Through IRS1/GSK3β/Wnt3a‐β‐Catenin Pathway

**DOI:** 10.1002/brb3.70056

**Published:** 2024-09-30

**Authors:** Kai‐Xin Zhang, Ning Sheng, Peng‐Li Ding, Ji‐Wei Zhang, Xiang‐Qing Xu, Ya‐Han Wang

**Affiliations:** ^1^ First College of Clinical Medicine Shandong University of Traditional Chinese Medicine Jinan China; ^2^ Beijing University of Chinese Medicine East Hospital, Zaozhuang Hospital Zaozhuang China; ^3^ College of Traditional Chinese Medicine Shandong University of Traditional Chinese Medicine Jinan China; ^4^ School of Acupuncture‐Moxibustion and Tuina Shandong University of Traditional Chinese Medicine Jinan China; ^5^ Department of Neurology Affiliated Hospital of Shandong University of Traditional Chinese Medicine Jinan China

**Keywords:** Alzheimer's disease, central glucose metabolism, Danggui Shaoyao San, insulin receptor substrate 1 (IRS1)/glycogen synthase kinase‐3β (GSK3β)/Wnt3a‐β‐catenin pathways

## Abstract

**Introduction:**

Alzheimer's disease (AD) is a neurodegenerative disease characterized by Amyloid plaques and neurofibrillary tangles. We explored the potential mechanism by which Danggui Shaoyao San (DSS) modulates central glucose metabolism via the insulin receptor substrate 1 (IRS1)/glycogen synthase kinase‐3β (GSK3β)/Wnt3a‐β‐catenin pathway, thereby exerting protective effects on cognitive functions.

**Methods:**

In vitro, HT22 cells were induced with streptozotocin (STZ) to investigate the impact of GSK3β on pathway transduction. The active components in the DSS stock solution were validated using mass spectrometry. Subsequently, an AD model in C57BL/6J mice was established through STZ injection into both ventricles. The success of the model was validated behaviorally and pathologically. The Morris Water Maze (MWM) test, immunohistochemistry, Western blotting, quantitative reverse transcription‐PCR, and 18F‐fluorodeoxyglucose‐positron emission tomography (FDG‐PET) were employed to evaluate the influence of DSS on memory and pathological changes in AD.

**Results:**

The DSS stock solution, rich in active components, ameliorated the memory deficits in AD mice in the MWM. In vitro, GSK3β exhibited regulatory control over Wnt and β‐catenin, with GSK3β inhibition mitigating β‐amyloid and tau redundancies at protein and gene levels, facilitating signal transduction. In vivo, DSS impacted key targets in the IRS1/GSK3β/Wnt3a‐β‐catenin pathway, mitigated senile plaques resulting from amyloid β (Aβ) deposition and neurofiber tangles induced by tau hyperphosphorylation, and alleviated the decline in central glucose metabolism observed in FDG‐PET.

**Conclusions:**

Our findings suggest that DSS potentially confers cognitive protection by alleviating central hypoglycemia through the IRS1/GSK3β/Wnt3a‐β‐catenin pathway. This may serve as a promising therapeutic avenue for AD.

## Graphical abstract

In vitro, HT22 cells were induced with streptozotocin to investigate the impact of GSK3β on pathway transduction. The active components in the DSS stock solution were validated using mass spectrometry. Subsequently, an AD model in C57BL/6J mice was established through streptozotocin injection into both ventricles. The success of the model was validated behaviorally and pathologically. The Morris Water Maze test, immunohistochemistry, Western blotting, quantitative reverse transcription‐PCR, and 18F‐fluorodeoxyglucose‐positron emission tomography (FDG‐PET) were employed to evaluate the influence of DSS on memory and pathological changes in AD. DSS potentially confers cognitive protection by alleviating central hypoglycemia through the IRS1/GSK3β/Wnt3a‐β‐catenin pathway.

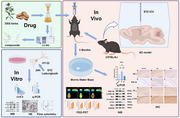

**Abbreviations**
AD: Alzheimer's disease
Aβ: β‐amyloid
DSS: Danggui Shaoyao San
FDG‐PET: 18F‐fluorodeoxyglucose‐positron emission tomography
GSK3β: glycogen synthase kinase‐3 β
IGF1R: insulin like growth factor 1 receptor
IR: insulin receptor
IRS1: insulin receptor substrate 1
MCI: mild cognitive impairment
NFTs: neurofibrillary tangles
sAPPα: APP‐cleaved fragment
STZ: streptozotocin

## 1 Introduction

Alzheimer's disease (AD) is the main body of dementia, characterized by cognitive impairment and memory loss (Selkoe and Hardy 2016). Due to the aging of the population and the slow development of drugs, AD puts a heavy burden on families and society. According to the *China Alzheimer's Disease Report 2024*, the number of existing cases of AD and other dementias in China in 2021 has reached 1.69 million. First‐line treatment drugs acetylcholinesterase inhibitors and excitatory amino acid receptor antagonists provide only partial relief. The therapeutic efficacy of monoclonal antibodies targeting amyloid beta clearance is still controversial (Tan et al. 2014). Therefore, potential therapeutic targets and drugs need to be continuously explored.

In addition to amyloid β (Aβ) aggregation and neurofibrillary tangles (NFTs) formation, which are considered major pathological changes in AD, the energy demands in the brain for neuronal function, axon transport, and neurotransmitter synthesis require glucose metabolism, which has implications for maintaining homeostasis related to neurodegenerative changes in AD (Butterfield and Halliwell 2019). Regulating glycogen synthase kinase‐3β (GSK3β)/Wnt3a/β‐catenin pathway and enhancing central glucose metabolism may actively prevent cognitive decline and protect the brain from tau and Aβ toxicity (Sellers et al. 2018; Nusse and Clevers 2017; Cisternas et al. 2019; Hwang et al. 2019).

Autophosphorylation of insulin receptors (IRs) can lead to activation of IR substrate 1 (IRS‐1) under the regulation of insulin (DaRocha‐Souto et al. 2012). GSK3β can promote the degradation of IRS‐1 proteasome under insulin resistance conditions, thereby controlling the stability and activity of the IRS‐1 (Hurtado et al. 2012). Studies have shown that increased GSK3β activity is directly related to Aβ deposition, tau hyperphosphorylation, and synaptic damage (Martinez and Perez 2008; Kremer et al. 2011). The increase of GSK3β activity in AD mouse brain is correlated with insulin and Wnt signaling (Inestrosa and Arenas 2010). After activation of the typical Wnt/β‐catenin signaling pathway, β‐catenin translocations to the nucleus and induces downstream gene expression (Nusse and Clevers 2017). The inhibited form of GSK3β(GSK3β(Ser9)) promotes downstream gene expression of β‐catenin. It is currently believed that the GSK3β/β‐catenin/Wnt signal can promote AD in the “on” state (McLeod et al. 2018).

Mice injected with streptozotocin (STZ) in the lateral ventricle can simulate AD central glucose metabolism and cognitive impairment, which is a mature AD model (Kim and Cho 2020). Danggui Shaoyao San (DSS) has shown potential in the clinical treatment of AD (Qin et al. 2021). However, its mechanism needs to be further clarified. Therefore, we hypothesized that DSS can improve the cognitive dysfunction caused by central glucose metabolism disorder, and regulate the glucose metabolism‐related pathway.

## 2 Materials and Methods

### 2.1 Animals and Molding

Forty‐five six‐week‐old male C57BL/6J mice (Beijing Vital River Laboratory Animal Technology Co. Ltd., Beijing, China, license number [SCXK(Zhejiang)2021‐0006]) with a weight range of 22–25 g were employed. The mice were housed in the SPF Animal Feeding Room (SYXK [Lu] 20220009), maintained at controlled conditions (20–22°C, relative humidity 60%–70%, light cycle 7:00–19:00).

**FIGURE 1:**
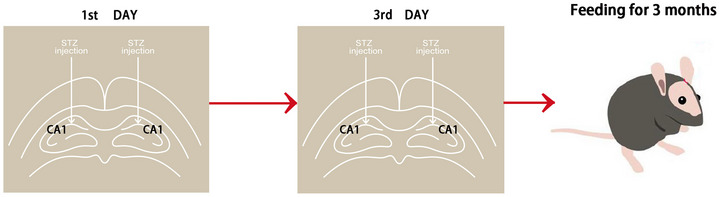
Illustration depicting the in vivo modeling process.

The molding method is consistent with the previous research (Qin et al. 2021). After fasting for 12 h, the mice were given abdominal anesthesia with Avertin (0.1 mL/10 g), and the anesthetized mice were fixed on a stereoscope. The bone was drilled with a cone frequency at 1.0 mm behind the fontan and 1.5 mm next to the sagittal suture. When the dura was exposed, the needle was inserted 2 mm vertically from the surface of the brain, and 5 µL liquid was slowly injected in 5 min, and the incision was sutured after injection. Fifteen mice were randomly selected to be injected with artificial cerebrospinal fluid (equivalent to STZ solution) in bilateral lateral ventricles on the 1st and the 3rd day as the control group, and the other 30 mice were injected with STZ solution (dissolved in artificial cerebrospinal fluid, 6 mg/mL, 3 mg/kg) in bilateral lateral ventricles on the 1st and the 3rd day as the AD model. The model production process is depicted in Figure 1. After 3 months, the control, AD, and DSS groups comprised 14, 14, and 13 mice, respectively.

### 2.2 Cells

HT‐22 cells (mouse hippocampal neurons; CL‐0595; ProCell, Wuhan, China) were cultured in a 5% CO_2_ incubator at 37°C using Dulbecco's modified Eagle medium (DMEM) containing 10% fetal bovine serum and 1% streptomycin. Upon reaching 80% cell density, cell passaging was performed, and third‐generation cells were used. To simulate an AD model in vitro, HT22 cells were treated with 10 mM STZ cell culture medium (26.522 mg STZ dissolved in 10 mL complete medium) (HY‐13753, MedChemExpres, Shanghai, China). Laduviglusib (CT9902, MedChemExpres) was employed to inhibit GSK3β. The cells were categorized into HT22 (H), HT22 + STZ (S), and HT22 + STZ + Laduviglusib (7 nM) (L) groups.

### 2.3 Reagents and Equipment

Detailed information is provided in Supporting Information Appendix A.

### 2.4 Drug Preparation and Ingredient Identification

Daily dose of DSS for human use includes Danggui (9 g), Shaoyao (48 g), Chuanxiong (10 g), Poria (12 g), Zexie (24 g), and Baishu (12 g). The daily dose of each mouse = human dose (g)/60 kg × 9.1 × 0.02 (converted according to the adult weight of 60 kg). Therefore, the daily DSS dose for 15 mice included Danggui (0.4 g), Shaoyao (2.2 g), Chuanxiong (0.46 g), Poria (0.55 g), Zexie (1.1 g), and Baishu (0.55 g). DSS non‐decocting granules (batch number: 2306500101) were dissolved in double steaming water (the volume of double steaming water was calculated according to 0.1 mL/10 g mouse body weight), preserved at 4°C, and administered once a day by intragastric administration. The control group was given the same amount of double steaming water once a day. The active components of the DSS stock solution were determined using ultra‐high‐performance liquid chromatography (LC)‐Q extraction mixed quadrupole orbital well mass spectrometry (MS) (Li, Ma et al. 2022), and three samples were collected.

### 2.5 Hematoxylin–Eosin (HE) Staining

Cells were digested with pancreatic enzyme, and the cell concentration was adjusted to approximately 1 × 10^5^/mL. The HT‐22 cells (mouse hippocampal neurons) were on a cover slide in a 6‐well plate. Following cell adhesion, different concentrations of STZ were administered for 24 h. HE staining process was consistent with previous studies (Hwang et al. 2019).

### 2.6 Cell Counting Kit‐8 (CCK‐8)

HT22 cells were harvested during the logarithmic growth phase, and the cell suspension density was adjusted to 1.0 × 10^4^ cells/mL. Subsequently, 100 µL was inoculated into each well of 96‐well plates, with 6 multiple wells designated for each group based on STZ concentrations of 0, 5, 10, 15, 20, 25, and 50 mM. A blank group containing only the medium was established, resulting in a total of eight groups. CCK‐8 process was consistent with previous studies (Wang, Huang, Hu et al. 2022).

### 2.7 Flow Cytometry

To assess the apoptosis rate in HT‐22 cells, an Annexin V‐PE Apoptosis Assay Kit (MA0429, Meilune, China) was employed. Flow cytometry was consistent with previous studies (Wang, Huang, Hu et al. 2022).

### 2.8 Morris Water Maze (MWM)

The operation process was based on the previous research (Qin et al. 2021). MWM test (MWM) was carried out in a quiet room. The water is injected into the pool (22°C ± 2°C), and the depth is 1 cm above the platform. Four quadrants on the inner wall of the pool are visual signs with different graphics and colors. The circular pool was divided into four quadrants, among which there was a platform in the first quadrant. The mice were trained for 3 days, with the maximum incubation period set to 60 s. On the 3 days of training, the mice were first guided to stand on the platform for 10 s to help the mice recognize and remember the platform's position, and then entered the water from the second and third quadrants facing the pool wall. The swimming distance for the mice to find the hidden platform was recorded within 60 s, and the route the mice traveled was recorded. If the mice could not find the platform within 60 s, it was placed on the platform for 10 s to help it remember the platform's position.

In the withdrawal experiment, mice were put into water from the third quadrant for 60 s, and the numbers of crossing the platform were recorded, and the residence time the mice remained in the target quadrant was recorded. The experiment was repeated five times for each group.

### 2.9 Fresh Tissue Specimens

After sacrificing the mice, hippocampal tissue was dissected on ice and transferred to an Eppendorf tube, then placed in a liquid nitrogen tank at −180°C for preservation. Each group had three samples.

### 2.10 Immunohistochemistry

The implementation process is consistent with the previous literature (Qin et al. 2021). The whole‐brain tissue of each mouse was removed and soaked in a 4% paraformaldehyde fixation solution after perfusion fixation. Then, embedding was carried out, and the slices were dehydrated and made transparent (thickness was 4 µm). After the slices were baked in an incubator at 60°C for 1 h, gradient alcohol dewaxing was performed; then, the antigen repair box was filled with a citrate repair solution, and the slices were inserted for microwave repair. After repair, each section was washed with PBS. The endogenous peroxidase blocker of the ultrasensitive SP rabbit (KTI‐9707, MBX) was added and left to stand for 10 min; then, the brain slices were cleaned as before.

After incubation by dropwise addition of a nonspecific dyeblocker for 10 min, the droplets were removed directly, and the primary antibody was added for incubation overnight. On the second day, the wet box was removed and placed at room temperature for 1 h, washed with PBS, and combined with the biotin‐labeled sheep anti‐rabbit IgG polymer. After washing, streptomycin anti‐biotin protein–peroxidase was added. After DAB color development, hematoxylin was reapplied. After gradient alcohol dewatering, the film was sealed. The stained area in the hippocampal CA1 region was measured using Slide Viewer software at different magnifications (15× and 50×) for each group of four samples.

### 2.11 Western Blotting (WB)

The implementation and data analysis were consistent with those in the previous literature (Qin et al. 2021). Each group was repeated three times. Brain tissue was added to RIPA tissue/cell lysate (R0010, Solarbio) at a ratio of 1:9 and left on ice for half an hour. Then, the brain tissue was centrifuged at 4°C for 15 min at a speed of 12,000 r/min. The bicinchoninic acid (BCA) method was used to measure the protein concentration and calculate the amount of protein loading. The glue concentration was determined according to the molecular weight of the protein; then, the separation of protein by SDS‐polyacrylamide gel electrophoresis (SDS‐PAGE), electron transfer, and sealing was carried out to incubate the primary antibodies overnight. On the second day, the primary antibody was shaken for 1 h and then cleaned with TBST for 10 min × 3 times. Then, the HRP‐conjugated secondary antibodies were incubated for 1 h and cleaned. ECL was performed for visualization. Each target protein was scanned across three bands, and the gray values of the target band and GAPDH were analyzed. GAPDH was taken as an internal reference, and ImageJ was used to calculate the gray value of the target band/GAPDH, after which additional statistical analysis was performed.

### 2.12 Quantitative Reverse Transcription‐PCR (q‐PCR)

Sample preparation, total RNA extraction of a tissue, RNA inversion in vitro, and q‐PCR were performed following the protocol described in our previous research (Qin et al. 2021). The primer sequences are listed in Supporting Information Appendix B. The mRNA levels of GAPDH were used as normalization controls. The relative quantitative method of 2^−∆∆^ *^Ct^* was used to determine the relative expression levels of each target gene.

### 2.13 18F‐Fluorodeoxyglucose‐Positron Emission Tomography (FDG‐PET)

Three mice from each group were randomly selected for FDG‐PET. After injecting FDG through the tail vein, the mice were continuously anesthetized with isoflurane for 50 min. Subsequently, the mice were positioned for PET/CT collection using NMSoft‐AIAC software (CT: voltage 50 kV, current: 0.5 mA, CT exposure time: 80 ms, PET collection time: 10 min). Reconstruction parameters for CT included a reconstruction algorithm: FDK, rebuild matrix: 512 × 512, pixel size: 0.1367, section thickness: 0.18. PET reconstruction algorithm: ListMode, rebuild matrix: 140 × 140, number of iterations: 40, pixel size: 0.5 mm, and section thickness: 0.8 mm. Image processing was performed using PMOD software, and the PFUS module was applied to process the data, capturing statistical quantitative values for each brain area along with corresponding images.

### 2.14 Statistical Analysis

Data analysis and visualization were conducted using GraphPad Prism 9.0 and SPSS 19.0. For experiments with three replicates, all data are presented as mean ± SD. Pursuant to the assumptions of a normal distribution and homogeneous variances, one‐way ANOVA between groups was selected, and an LSD *t* test was used to compare pairs. Conversely, the Kruskal–Wallis *H* test was used to compare multiple samples. Statistical significance was set at *p* < 0.05 and *p* < 0.01.

## 3 Results

### 3.1 STZ‐Induced Apoptosis of HT‐22 Cells

**FIGURE 2:**
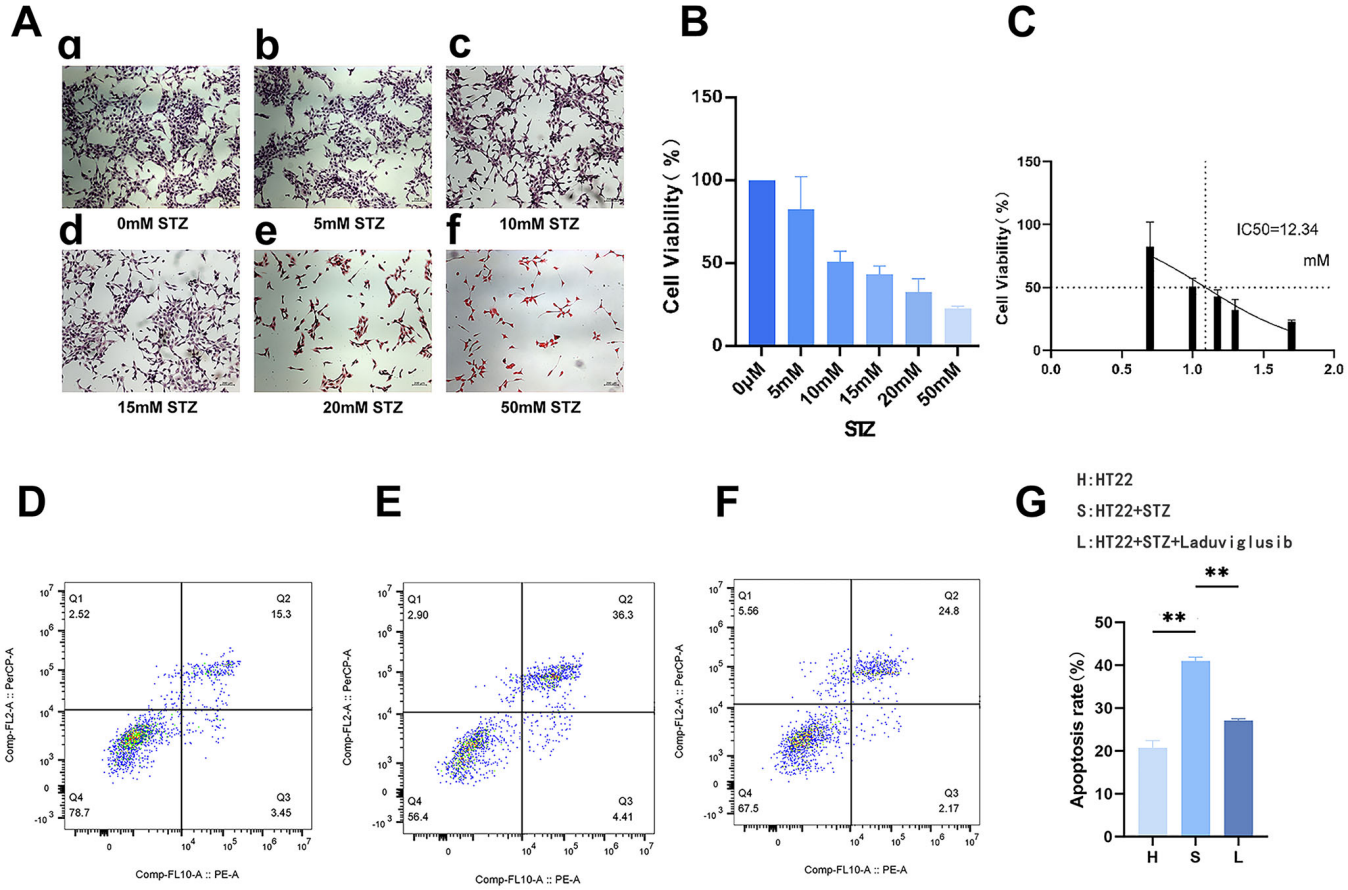
Effects of STZ on HT‐22 apoptosis. (A) Hematoxylin and eosin (HE) staining; (B) CCK‐8 measurement of STZ on HT‐22 24‐h cell survival rate; (C) STZ intervention in HT‐22 24 h IC50 calculation; (D) apoptosis rate of HT‐22 cells in the H group detected by flow cytometry; (E) apoptosis rate of HT‐22 cells in the S group detected by flow cytometry; (F) apoptosis rate of HT‐22 cells in the L group detected by flow cytometry; (G) statistical comparison of three groups, **p < 0.01. CCK‐8, cell counting kit‐8; STZ, streptozotocin.

HE staining was performed 24 h after treating HT‐22 cells with different concentrations of STZ. The findings revealed a decrease in HT‐22 cell density with increasing STZ concentration. Approximately 50% cell density reduction occurred at approximately 15 mM STZ. Concurrently, alterations in cell morphology, including neuronal cell body shrinkage, reduction in synapse size, and diminished neuronal connections, were observed (Figure 2A).

CCK‐8 results (Figure 2B) indicated a gradual decrease in cell survival rate with increasing STZ concentration, yielding an IC50 value of 12.34 mM (Figure 2C). To enhance HT‐22 cell survival, 10 mM STZ concentration was selected for subsequent experiments. Flow cytometry was then employed to evaluate the impact of STZ and Laduviglusib (a GSK3β inhibitor) on HT‐22 apoptosis (Figure 2D–F). An increased HT‐22 apoptosis rate was observed 24 h post‐STZ induction, which improved following Laduviglusib intervention (Figure 2G), suggesting that GSK‐3β inhibition could mitigate HT‐22 apoptosis, thereby potentially ameliorating AD symptoms.

### 3.2 Inhibition of GSK3β In Vitro Leads to Upregulation of the Wnt‐β‐Catenin Pathway and Alteration of p‐Tau

**FIGURE 3:**
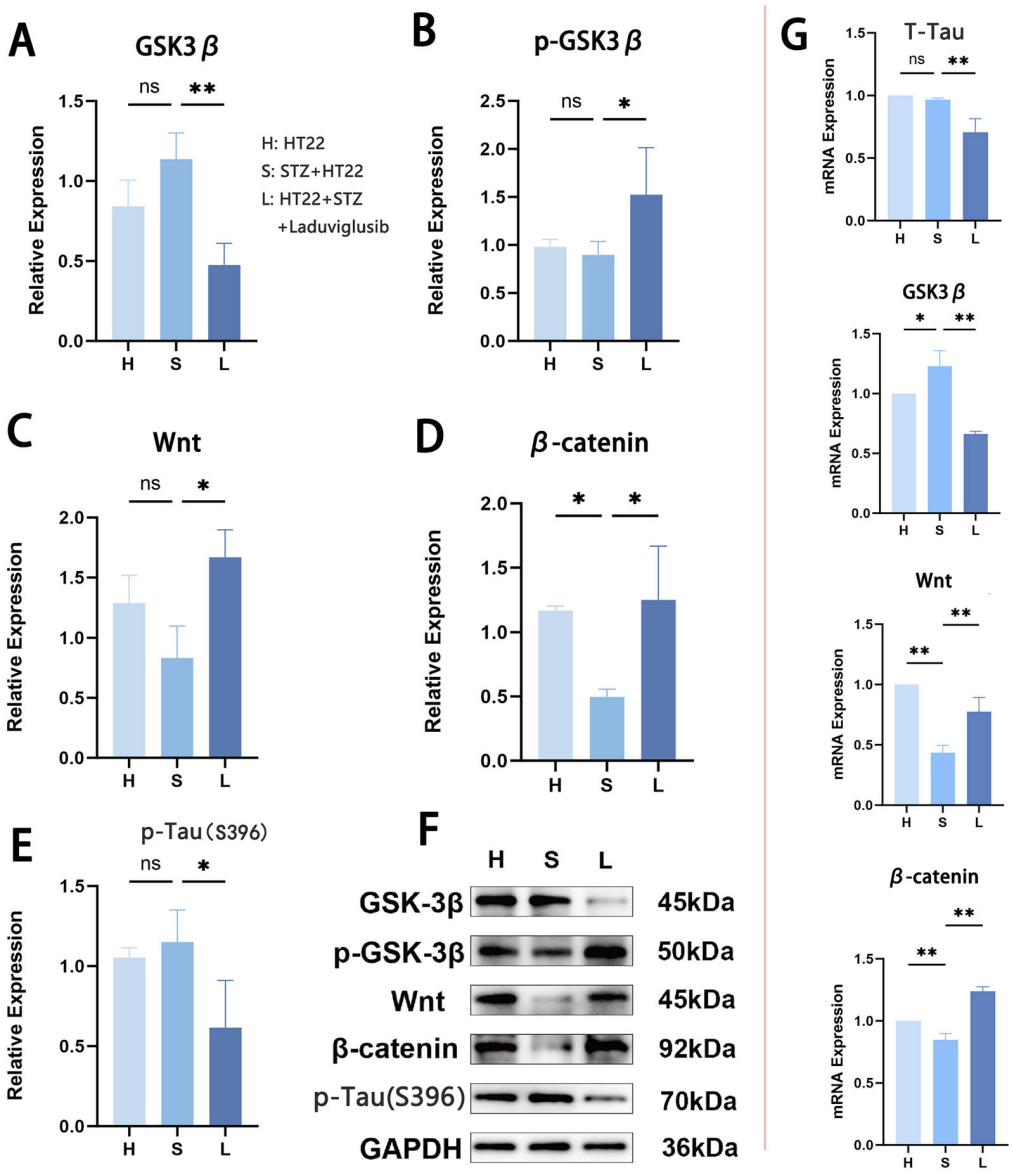
Inhibition of GSK3β in vitro leads to upregulation of Wnt‐β‐catenin pathway and change of p‐tau. (A) Western blotting (WB) showing Laduviglusib‐mediated inhibition of GSK3β expression; (B–D) WB showing increased expression of p‐GSK3, Wnt, and β‐catenin when GSK3β is inhibited; (E) WB showing decreased p‐tau expression when GSK3β is inhibited; (F) WB showing expression of different proteins; (G) reverse transcription‐quantitative polymerase chain reaction (Rt‐qPCR) showing increased relative expression of GSK3β mRNA in the S group and decreased expression when GSK3β is inhibited, with a similar trend observed for tau mRNA. The mRNA relative expression of Wnt and β‐catenin showed the opposite trend (*p < 0.05, **p < 0.01). GSK3β, glycogen synthase kinase 3β.

We utilized an STZ‐induced HT22 cell model to investigate the effect of inhibited GSK3β expression on the Wnt pathway. WB results revealed no difference in GSK3β and P‐GSK3β expression between HT22 cells and STZ‐treated HT22 cells. However, GSK3β expression decreased in STZ‐HT22 cells after GSK3β inhibitor treatment (**p < 0.01), whereas p‐GSK3β expression increased (*p < 0.05) (Figure 3A,B), indicating reduced GSK3β activity by Laduviglusib. Subsequently, we assessed Wnt and β‐catenin expression (Figure 3C,D). Although no difference in Wnt expression was noted between groups H and S, it increased following GSK3β inhibition along with β‐catenin. Notably, β‐catenin expression in STZ‐treated HT22 cells was lower than in the untreated H group (*p < 0.05). Given the regulatory effects of GSK3β and Wnt on tau phosphorylation, we evaluated p‐tau expression in these cells (Wang, Tanokashira et al. 2019; McLeod et al. 2018). Results indicated that STZ did not elevate p‐tau expression but decreased it in STZ‐HT22 cells post GSK3β inhibition (*p < 0.05) (Figure 3E). These protein expression patterns were reflected in the WB results (Figure 3F). RT‐qPCR analysis demonstrated that relative tau (*MAPT*) mRNA expression in group S did not surpass that in group H (Figure 3G). However, GSK3β expression decreased following Laduviglusib‐mediated inhibition (**p < 0.01), suggesting the regulatory role of GSK3β on tau. Moreover, relative mRNA expression levels of Wnt and β‐catenin increased post GSK3β inhibition (**p < 0.01), indicating the regulatory effect of GSK3β on Wnt and β‐catenin.

### 3.3 Determination of DSS Components

**FIGURE 4:**
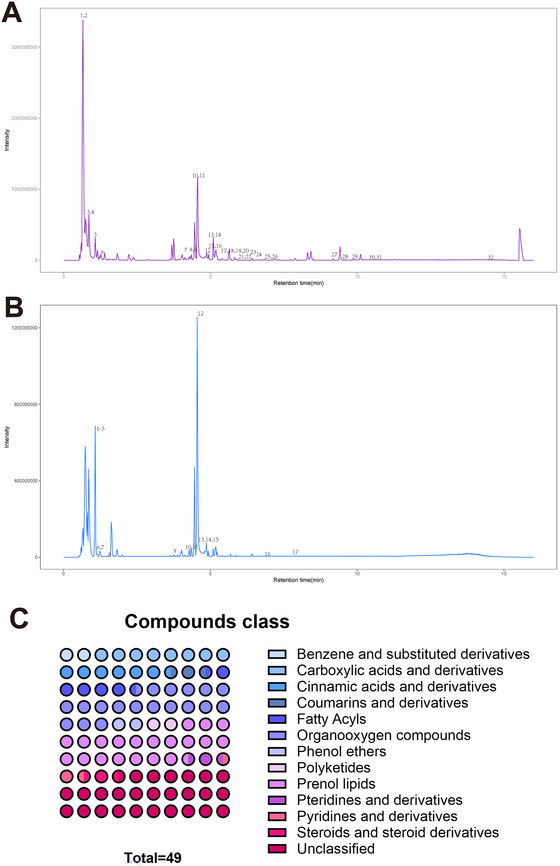
Composition of DSS determined using LC–MS. (A) Base peak chromatogram (BPC) in cationic mode; (B) BPC in anionic mode; (C) compounds class. A total of 49 components are divided into 13 categories, each color representing a category, distributed in percentage on a 10 × 10 dot chart. DSS, Danggui Shaoyao San.

Forty‐nine components were obtained by LC–MS; specifically, 32 components were detected in cationic mode (Figure 4A), and 17 metabolites were detected in anionic mode (Figure 4B) (Supporting Information Appendix B and C). Among the 40 compounds (Figure 4C), the 3 compounds with the highest proportions were prenol lipids (20.41%), organooxygen compounds (18.37%), and carboxylic acids and derivatives (8.16%). Paeoniflorin and albiflorin in paeoniflorin, and ferulic acid in *Angelica sinensis*, were detected. Other active ingredients, such as myristic acid, glycyrrhetinic acid, stachyose, and ligustroside, were also identified.

### 3.4 DSS Has a Cognitive Protective Effect on Bilateral Intraventricular Injection of STZ (STZ‐ICV) Mice

**FIGURE 5:**
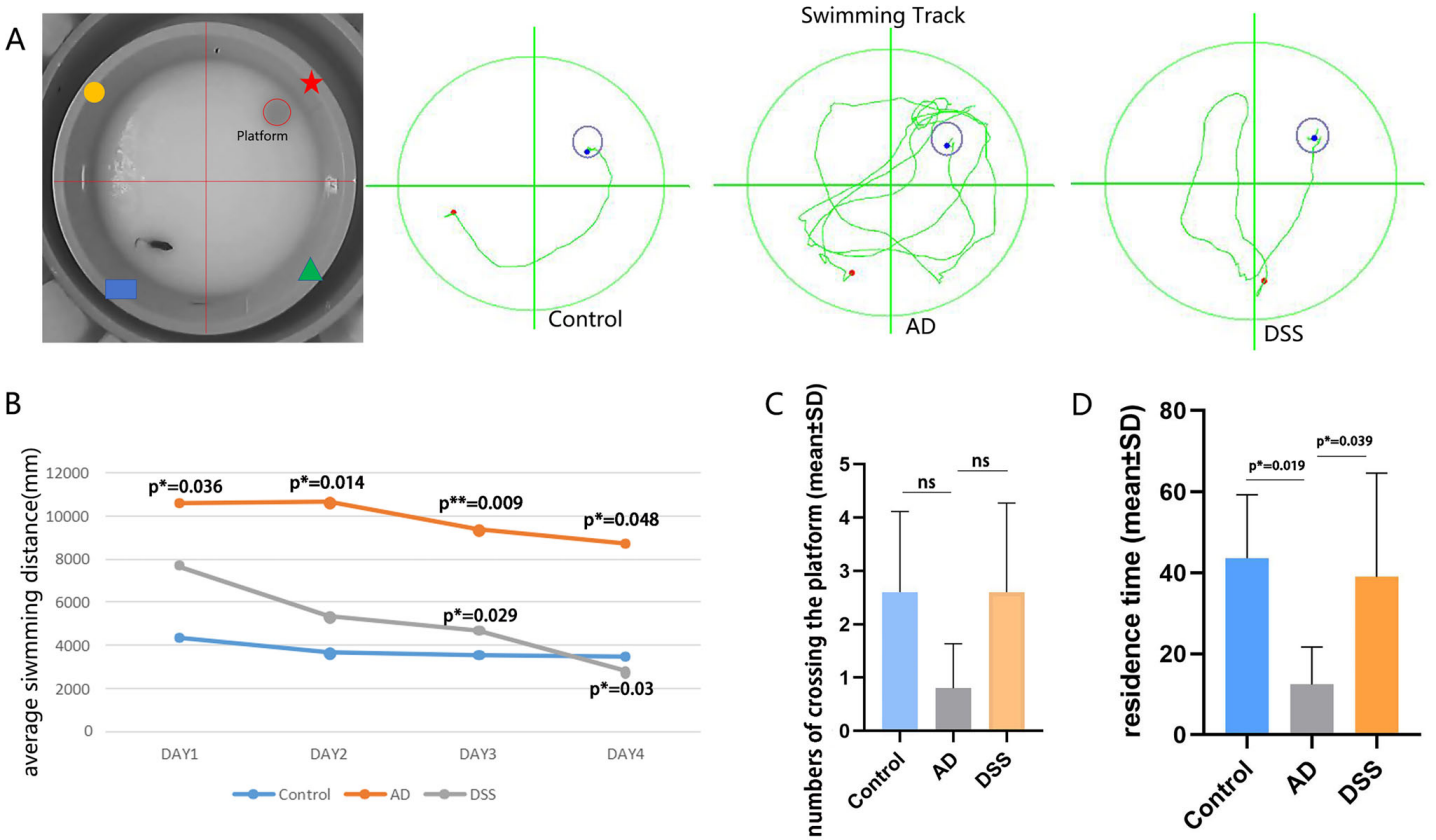
Morris Water Maze. (A) Layout of the water maze and swimming tracks of the three groups of mice; (B) average swimming distance of the three groups of mice during the first 4 days; (C) Number of times the three groups of mice crossed the platform during the withdrawal period; (D) residence time of the three groups of mice in the quadrant where the platform was located during the withdrawal period (*p < 0.05). DSS, Danggui Shaoyao San.

The layout of the water maze and swimming tracks of the three groups of mice recorded using the instrument are displayed in Figure 5A. In the positioning navigation test (Figure 5B), the average swimming distance of the three groups shortened with an increase in training time. The DSS group shortened at the fastest rate, whereas the control group did not decrease before and after training. We observed a statistically significant difference between the control and AD groups on Day 1 (*p < 0.05), and the average swimming distance of the AD group was consistently higher than that of the control group (*p < 0.05, **p < 0.01). In the first 2 days of training, the average swimming distance of the DSS group was shorter than that of the AD group, but the difference was not statistically significant. On the third day, the average swimming distance of the DSS group was different from that of the AD group (*p < 0.05), and this difference persisted until the fourth day (*p < 0.05).

After the platform was removed, the residence time of the AD group was shorter than that of the control group in the quadrant where the platform was located (*p < 0.05), and that of the DSS group was higher than that of the AD group (*p < 0.05) (Figure 5D). However, we observed no statistically significant difference among the three groups in terms of the number of crossings on the platform (Figure 5C).

### 3.5 DSS Affects the Expression of IRS1/GSK3β/Wnt‐β‐Catenin Pathway

**FIGURE 6:**
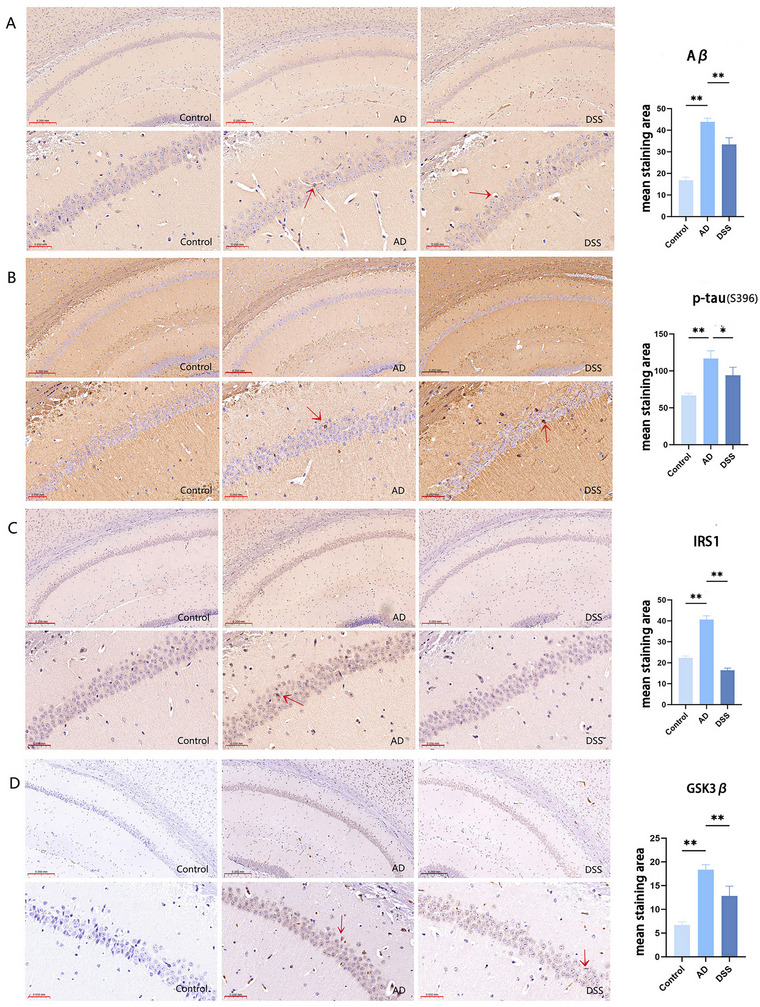
Immunohistochemical results. (A) Aβ deposited in the CA1 region of the hippocampus, and the brown color indicates positive staining (red arrow); (B) Hyperphosphorylated tau deposited in the hippocampal CA1 region with positive brown staining (red arrow); (C) IRS1 is brown stained in the hippocampal CA1 region (red arrow); (D) GSK3β is brown stained in the hippocampal CA1 region (red arrow) (bar = 200 µm, bar = 50 µm) (*p < 0.05, **p < 0.01). Aβ, amyloid β; DSS, Danggui Shaoyao San; GSK3β, glycogen synthase kinase 3β; IRS1, insulin receptor substrate 1.

**FIGURE 7:**
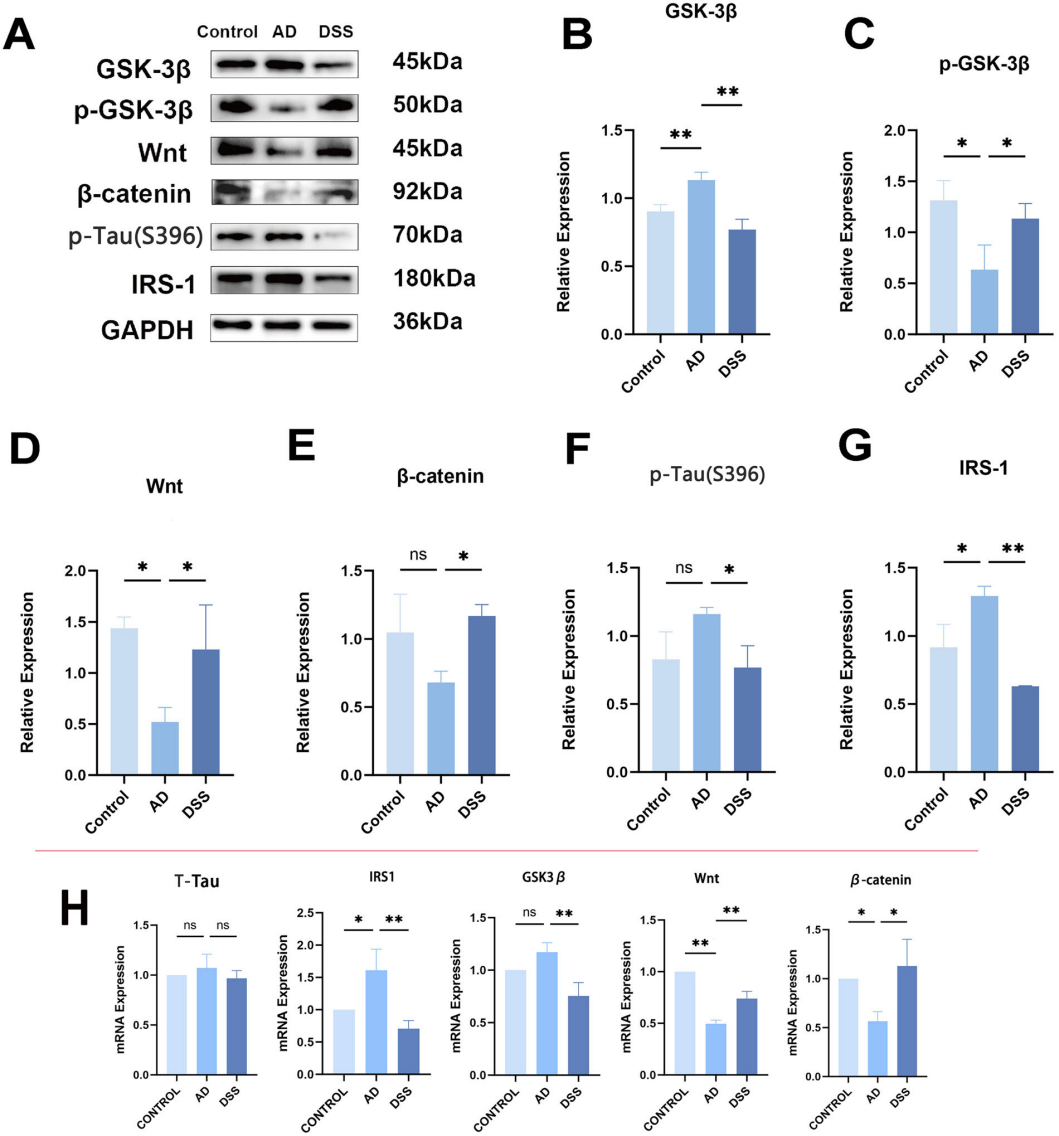
Western blotting and RT‐qPCR results. (A–G) Relative expression of pathway key proteins; (H) mRNA relative expression of pathway key proteins (*p < 0.05, **p < 0.01). DSS, Danggui Shaoyao San.

Immunohistochemical results revealed that the levels of Aβ deposition and p‐tau in the AD group were higher than those in the control group (**p < 0.01), and the expression levels of these two markers in the brains of DSS‐treated mice were lower than those in the AD group (*p < 0.05, **p < 0.01) (Figure 6A,B). However, WB showed that there was no statistically significant difference in the increase in p‐tau expression levels in the brains of AD mice compared to that in the control group (Figure 7F), suggesting that hyperphosphorylated tau was not the first to appear in the pathological evolution of AD. However, after DSS intervention, p‐tau expression increased (*p < 0.05), indicating that DSS can improve this pathological marker.

After identifying the regulatory role of GSK3β on the Wnt pathway, we assessed the effect of DSS on the IRS1/GSK3β/Wnt‐β‐Catenin pathway. Immunohistochemical and WB results revealed that the expressions of IRS‐1 and GSK3β in the AD group were both increased compared with those in the control group (*p < 0.05, **p < 0.01) and decreased after DSS treatment (**p < 0.01) (Figures 6C,D and 7B,G). The expression of p‐GSK3β exhibited the opposite trend (*p < 0.05) in WB (Figure 7C). Additionally, WB demonstrated that, with the increase of GSK3β, the expression of Wnt and β‐catenin in the AD group was lower than that in the control group; however, the decrease of β‐catenin did not differ from that in the control group (*p < 0.05, *p* > 0.05) (Figure 7D,E). Following DSS treatment, the expression levels were higher than those in the AD group (*p < 0.05). This may be attributed to the inadequate reduction in Wnt caused by STZ in vivo to fully impede β‐catenin expression, whereas DSS can augment β‐catenin levels. RT‐qPCR results demonstrated that after DSS intervention, the mRNA relative expression levels of IRS1 and GSK3β decreased compared with those in the AD group (**p < 0.01), whereas the relative mRNA expression levels of Wnt and β‐catenin increased compared with those in the AD group (*p < 0.05, **p < 0.01), thereby confirming the regulatory role of DSS on the pathway at the genetic level (Figure 7H).

### 3.6 DSS Improves Central Glucose Metabolism in STZ‐ICV Mice

**FIGURE 8:**
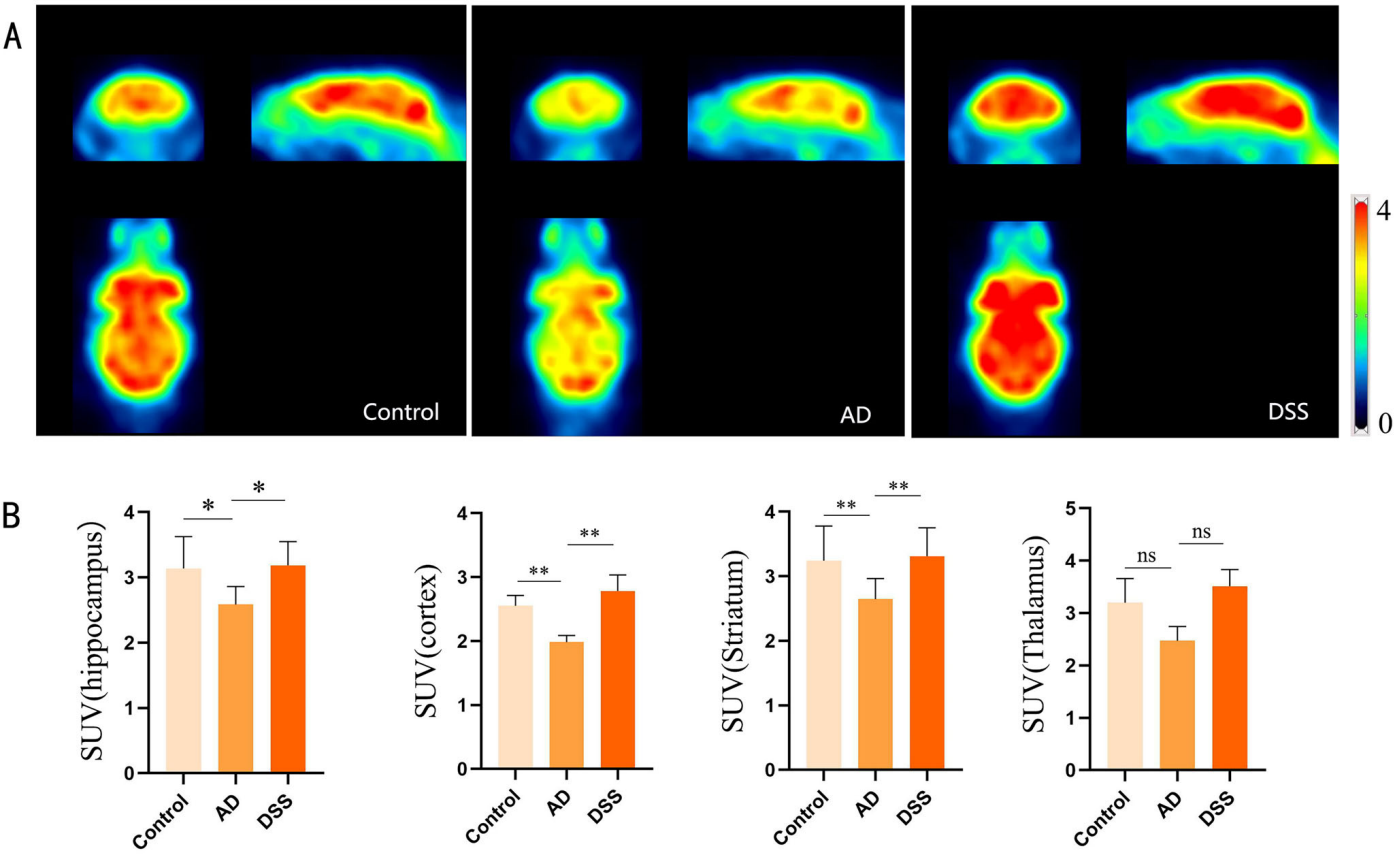
18F‐Fluorodeoxyglucose‐positron emission tomography (FDG‐PET) results. (A) Whole‐brain glucose metabolism imaging of mice; (B) comparison of glucose metabolism levels in the hippocampus, cortex, striatum, and thalamus of mice (*p < 0.05, **p < 0.01). DSS, Danggui Shaoyao San.

In a cohort study of mild cognitive impairment (MCI), patients with MCI exhibited reduced levels of glucose metabolism in the brain, implying that a disorder in central glucose metabolism contributes to cognitive impairment in AD (An et al. 2018). PET was employed to assess glucose metabolism in the hippocampus, cortex, striatum, and thalamus of mice in each group (Figure 8A). The results revealed that glucose metabolism levels in the three brain regions of the AD group were lower than those in the control group, with the exception of the thalamus. Following DSS treatment, glucose metabolism levels in the three brain regions increased. Although the thalamic region results did not exhibit a statistical difference between the groups, they displayed a similar trend (*p < 0.05, **p < 0.01) (Figure 8B).

## 4 Discussion

However, the primary focus of AD research has been on the hallmark features—Aβ and tau—rectifying these elements might not entirely alleviate cognitive impairment. A growing body of research now directs attention to an earlier pathological manifestation in patients and animal models of cognitive impairment, specifically the disruption of glucose metabolism in the central nervous system. AD mice established by intraventricular injection of STZ can reflect central insulin resistance and simulate the decline of central glucose metabolism and cognitive deterioration in early AD without peripheral insulin resistance (Qin et al. 2021). Insulin, a pivotal neurohormone, plays a crucial role in regulating central glucose metabolism and cognitive function (Duarte et al. 2018). IRS‐1 is a direct substrate of the insulin‐sensitive IR and insulin‐like growth factor‐1 receptor (IGF1R). The IRS1‐mediated insulin signaling pathway plays a crucial role in the regulation of growth, glucose, and energy metabolism. Insulin signaling exerts its biological role by modulating IRS proteins through the phosphorylation of serine (Ser) and threonine (Thr) residues. Additionally, IRS‐1 can undergo detachment from the control of insulin and IGF1R, undergoing phosphorylation on Ser and Thr residues. Numerous studies have elucidated the relationship between IRS1 and GSK3β (Evangelisti et al. 2020). GSK3β serves as a pivotal downstream molecule in the insulin pathway and a key regulator of tau phosphorylation and Aβ deposition (Cisternas et al. 2019). Although the extensive phosphorylation of IRS1 upstream of insulin signaling at specific Ser sites is reportedly not associated with Aβ in models of age‐related memory deficits or peripheral glucose metabolism abnormalities, (Hernandez, Lucas, and Avila 2013) studies on retinal damage in diabetes have confirmed that the impaired IRS1 signaling pathway activates GSK3β. Subsequently, acting on the insulin signaling pathway, the increased activity of GSK3β accelerates the hyperphosphorylation of p‐tau at the AD‐associated phosphorylation site, leading to synaptic loss in the diabetic retina (Qin et al. 2021).

Moreover, IRS‐1 and GSK3β may also mediate myelin and synaptic damage. Highly phosphorylated tau impairs mitochondrial transport and reduces mitochondrial activity in a GSK3β‐dependent manner, directly impairing cognitive function (Chen et al. 2024). The APP‐cleaved fragment (sAPPα) interacts with IGF1 and IRs in physiological settings (Maesako et al. 2017). This interaction may promote neuronal survival through the phosphorylation and inhibition of GSK‐3β (Karimian et al. 2019). The activation of GSK‐3β elucidates several features of AD, such as age spot accumulation, memory loss, induced inflammation, synaptic damage, and neurodegeneration through the activation of astrocytes and increased tau phosphorylation (Jimenez et al. 2011; Llorens‐Martín et al. 2014). GSK3β, also known as tau kinase I, plays a pivotal role in Aβ and p‐tau redundancy (An et al. 2018). During the pathological process of AD, GSK3β induces tau phosphorylation on a set of phosphorylated residues, leading to tau separation from microtubules, destabilizing the cytoskeleton, and forming neurotoxic oligomers, further impairing cell function. In our study, IRS1 expression increased in STZ‐HT22 cells, and p‐tau expression also increased compared to HT22 cells, suggesting a correlation between IRS1 and p‐tau. After GSK3β was inhibited, the expression of p‐tau in STZ‐HT22 cells reduced compared to the model group, thereby validating the regulatory effect of GSK3β on p‐tau.

Wnt signaling plays a pivotal role in neuronal development, synaptogenesis, axonal remodeling, and long‐term enhancement associated with Alzheimer's (McLeod et al. 2018). Wnt ligands secrete glycoproteins through lipid modification and, upon binding to cell surface receptors, trigger intracellular signaling pathways that regulate various biological processes such as the cell cycle, cell migration, and establishment of cell polarity (Liu et al. 2022). Wnt ligands trigger intracellular signaling pathways induced by the receptor complex, including both the typical Wnt/β‐catenin and atypical β‐catenin‐independent signaling pathways (Wang, Huang, Su et al. 2022). In this particular pathway, when classical Wnt ligands are absent, protein complexes, including GSK‐3β, are disrupted. The core link of Wnt signaling involves the inhibition of GSK3β by transcription cofactor β‐catenin, which shields it from proteasome degradation. In other words, the Wnt signal inhibits GSK3β, allowing β‐catenin to accumulate in the cytoplasm and subsequently translocate to the nucleus to mediate the transcription of specific genes, influencing the pathogenesis of AD (Zheng et al. 2017). The absence of the Wnt ligand also results in the phosphorylation of β‐catenin in the cytoplasm under the influence of GSK‐3β, leading to its degradation. Previous studies have identified impaired Wnt signaling as a commonality between age‐induced neurodegeneration and AD, specifically noting decreased expression of Wnt ligands and receptors, and increased activity of GSK‐3β (as a negative regulator of Wnt signaling), resulting in an overall downregulation of the Wnt pathway (Hou et al. 2019). Our cell studies affirm the impact of GSK3β on Wnt‐β‐catenin: When GSK3β expression is inhibited, its phosphorylated form increases, and the diminished activity of GSK3β improves the stability of the protein complex, leading to upregulation of the Wnt pathway.

GSK3β and Wnt signaling are not only related to tau hyperphosphorylation but also affect amyloid deposition (Norwitz et al. 2019). Some studies have suggested that Wnt signals can act at the gene level and influence the expression of β‐secretase and α‐secretase, resulting in the reduction of APP phosphorylation in amyloid protein production, thereby preventing the generation of Aβ (Pan et al. 2018). In our in vivo experiments, the expression of IRS1 and GSK3β increased, the expression of Wnt‐β‐catenin was downregulated, and the deposition of Aβ and phosphorylated tau increased in AD mice. This suggests the regulatory role of the IRS1/GSK3β/Wnt‐β‐catenin signaling pathway in Aβ and p‐tau at the in vivo level. This finding aligns with those of previous studies (Liu et al. 2022).

DSS is composed of Radix *A. sinensis*, Chinese herbaceous peony, Rhizoma Chuanxiong, Rhizoma Atractylodis, Rhizoma Alismatis, and Poria cocos. Studies have demonstrated its efficacy in addressing Aβ and neuronal degeneration in AD by regulating lipoprotein receptor‐associated protein‐1 and the receptor for advanced glycation end products (Yang et al. 2021). However, owing to the complex action networks of traditional Chinese medicine (TCM) compounds, more action targets require further exploration. We identified several of the most representative components of DSS, which have been shown to have therapeutic potential for AD. Paeoniflorin can inhibit iron death in PC12 cells and reduce oxidative stress damage, reduce iron death level in AD mice, reduce iron ion and MAD level in brain tissue, and thus improve the cognitive ability of AD mice (Zhai et al. 2023). In addition, paeoniflorin also had a protective effect on cerebral cortex inflammation and apoptosis in AD mouse models (Gu et al. 2016). Albiflorin has been found to reduce hippocampal Aβ levels in STZ‐induced cognitive impairment rats and significantly reduce serum insulin levels in rats (Ma et al. 2021). Albiflorin can also regulate the Nrf‐2/HO‐1/HMGB1/NF‐κB signaling pathway in the hippocampus to improve oxidative stress and inflammation (Ma et al. 2021). Ferulic acid can alleviate the oxidative stress damage of neurons (Kumar and Pruthi 2014). Ferulic acid improves Aβ neurotoxicity by several mechanisms, and pharmacological studies have revealed that Ferulic acid can regulate brain insulin signaling, Aβ accumulation, and synaptic plasticity in hyperinsulinemia rats, thus having a protective effect on the pathogenesis of AD (Singh et al. 2021). To further explore the therapeutic effects of DSS on AD, we administered DSS to STZ‐ICV mice. Previous studies have indicated that the AD mouse model with bilateral lateral ventricular injection of STZ can not only manifest Aβ and tau redundancy but also simulate the pathological changes of central insulin resistance in the early stage of AD, which holds significant value for the study of late‐onset AD (where the gene effect is not significant) (Wang, Wang et al. 2019). In this study, mice in the AD group exhibited an increase in the average swimming distance in the water maze test, a decrease in residence time in the platform quadrant, and an improvement in memory after DSS intervention. The number of age plaque deposits and NFTs in the brains of AD mice was higher than that in the control group and reduced after DSS intervention. The expressions of IRS1 and GSK3β decreased, and those of Wnt and β‐catenin increased in the brains of DSS‐treated mice. This suggests that the cognitive impairment of AD mice may be related to the insulin signaling pathway, and DSS intervention may reduce the accumulation of neuropathological products by regulating the IRS1/GSK3β/Wnt‐β‐catenin pathway, thus playing a cognitive protective role.

To further observe the effects of DSS on central glucose metabolism, we measured glucose metabolism in the brains of mice using 18F FDG‐PET. The AD autopsies showed that the basic activation of IRs in the brain was reduced, and the ability to maintain glucose and energy homeostasis of cells was impaired, thus triggering the occurrence of AD neurodegenerative changes (Steen et al. 2005). The loss of tau‐stable microtubule function may also reportedly lead to cerebral insulin resistance (Marciniak et al. 2017). Therefore, maintaining homeostasis of central glucose metabolism is a valuable direction for drug therapy. Reduced brain metabolism, as measured by FDG‐PET, has been used by clinical investigators to improve the diagnostic accuracy of AD and MCI, and attention has been paid to the correlation between brain metabolic rate and neuronal activity (An et al. 2018). Abnormal glucose metabolism in different brain regions can be used to assess different types of dementia (Kato et al. 2016; Moonga et al. 2017). In our study, STZ‐induced HT22 cells only showed an increase in the pathological markers of AD, with no statistical difference compared with the control group. However, STZ‐induced intracellular NFTs formed by tau hyperphosphorylation and extracellular age spots formed by Aβ deposition in the AD group increased compared with those of the control group, and the brain metabolic rate of the AD group was lower, as detected by PET. This suggests that the disorder of glucose metabolism in the brain may impair cognitive function earlier than the redundancy of Aβ and tau, further promoting the deterioration of AD. Multiple brain regions (cortex, hippocampus, striatum, and thalamus) of mice treated with DSS showed normalization of glucose metabolism, confirming the role of DSS in protecting central glucose homeostasis.

## 5 Conclusions

In our study, DSS improved the cognitive decline observed in AD mice, as evidenced by improved performance in the water maze and a reduction in the deposition of pathological products in their brains. These findings substantiate the efficacy of DSS. Experimental results, considering both protein and gene levels, suggest that DSS modulates the IRS1/GSK3β/Wnt‐β‐catenin pathway. This modulation appears to be linked to the ability of DSS to enhance glucose metabolism levels in the AD brain and alleviate the accumulation of Aβ and tau. However, owing to the lack of in vivo pathway interference in this study, there remains insufficient evidence to conclusively establish that DSS can impact the expression of GSK3β in AD and exert a cognitive protective effect. Additionally, the relationship between the IRS1/GSK3β/Wnt‐β‐catenin pathway and central glucose metabolism warrants further verification.

## Author Contributions

**Kai‐Xin Zhang**: project administration, writing–original draft. **Ning Sheng**: writing–review and editing, visualization. **Peng‐Li Ding**: visualization, formal analysis. **Ji‐Wei Zhang**: methodology, investigation. **Xiang‐Qing Xu**: conceptualization. **Ya‐Han Wang**: supervision, writing–review and editing, funding acquisition.

## Ethics Statement

All experimental procedures were approved by the Animal Ethics Committee of Shandong University of Chinese Medicine and carried out in accordance with ARRIVE guidelines (Animal Ethics: SDUTCM20230407002).

## Conflicts of Interest

The authors declare no conflicts of interest.

### Peer Review

The peer review history for this article is available at https://publons.com/publon/10.1002/brb3.70056.

## Supporting information



Supporting Information

Supporting Information

Supporting Information

Supporting Information

## Data Availability

The datasets used and/or analyzed during the current study are available from the corresponding author on reasonable request.
