# Supplementary material for: Danggui Shaoyao San Alleviates Early Cognitive Impairment in Alzheimer's Disease Mice Through IRS1/GSK3β/Wnt3a‐β‐Catenin Pathway
Source: Brain Behav. 2024 Sep 30;14(10):e70056. doi: 10.1002/brb3.70056 (PMC11440033; doi:10.1002/brb3.70056)
Supplement: Supplementary file 1 — Supporting Information [file BRB3-14-e70056-s002.docx]

**Reagents and equipment**

Reagents

Tribromoethanol (Batch No. A103417, Sigma-Aldrich, Darmstadt, Germany)

Anti-beta amyloid 1-42 antibody [mOC64] (1:1000, ab201060, Abcam, Shanghai, China)

Anti-Tau (phospho S396) antibody (1:1000, ab32057, Abcam, Shanghai, China)

Anti-GSK3beta antibody (1:5000, ab32391, Abcam, Shanghai, China)

Phospho-GSK-3β (Ser9) (D85E12) Rabbit mAb(1:1000, #5558, Shanghai, China)

Aniti-Wnt3a antibody [EPR21889] (1:1000, ab219412, Abcam, Shanghai, China)

Anti-beta Catenin antibody (1:5000, ab32572, Shanghai, China)

Animal non-immune serum (sheep) (SP KIT-B2, Fuzhou Maixin Biotech. Co., Ltd., Fuzhou, China)

Fast enzyme-labeled sheep anti-rabbit IgG polymer (KIT-5004, Fuzhou Maixin Biotech. Co., Ltd.)

Enhanced DAB Plus Kit (DAB-2031, Fuzhou Maixin Biotech. Co., Ltd.)

Solvents and chemicals used included anhydrous ethanol (Chengdu Chron Chemicals Co., LTD, Chengdu, China)

TRIzol (Ambion, Austin, Texas, USA)

Primers (Tsingke, Beijing, China)

Chloroform (Chengdu Guerda Rubber Industry Co., LTD, Chengdu, China)

No RNase water (Servare Biotech Inc., Wuhan, China)

Isopropyl alcohol (Chengdu Guerda Rubber Industry Co., LTD)

Equipments

S1000™ Thermal Cycler common PCR instrument (BIO-RAD, California, USA)

CFX Connect real-time quantitative fluorescent PCR instrument (BIO-RAD)

1. well quantitative PCR plate (Servare Biotech Inc)
2. PCR plate sealing film (LABSELECT, Beijing, China)

Chemiluminescence imaging system ChemiScope6100 (Shanghai Qinxiang Scientific Instrument Co., LTD, Shanghai, China)

H1 16KR table refrigerated high-speed centrifuge (Hunan Ke Cheng instrument Co., LTD, Hunan, China)

SC-3610 low-speed centrifuge (Anhui USTC ZONKIA scientific instruments Co., LTD, Anhui, China)

Handheld instantaneous centrifuge (SCLLOGEX, Shanghai, China), Vortex oscillator SCL-VS (SCLLOGEX)

Electrophoresis apparatus (164-5050) (BIO-RAD)

Pipette tips (Beijing Labgic Co., LTD, Beijing, China), Microsample feeder (Thermo scientific, Massachusetts, USA)

Handheld instantaneous centrifuge CF2800M (LABGIC, Beijing, China), Grinding machine (Servare Biotech Inc)

Sealing instrument (Servare Biotech Inc)

Animal PET/SPECT/CT-integrated machine (InliView-3000B; Novel Medical, Beijing, China)
