# Supplementary material for: Danggui Shaoyao San Alleviates Early Cognitive Impairment in Alzheimer's Disease Mice Through IRS1/GSK3β/Wnt3a‐β‐Catenin Pathway
Source: Brain Behav. 2024 Sep 30;14(10):e70056. doi: 10.1002/brb3.70056 (PMC11440033; doi:10.1002/brb3.70056)
Supplement: Supplementary file 2 — Supporting Information [file BRB3-14-e70056-s003.docx]

**Primers**

| Primers | Sequence | |
| --- | --- | --- |
| Gsk3β (*GSK3B*) F | CTACAGGGCACCAGAGTTGA | |
| Gsk3β (*GSK3B*) R | ACAATTCAGCCAACACACAGC | |
| Wnt3a (*WNT*) F | CGTACTTCAAGGTGCCGACA |  |
| Wnt3a (*WNT*) R | TTTCGGGGTTAGGTTCGCAG |  |
| Tau (*MAPT*) F | AAGTGTGGCTCGTTAGGGAA |  |
| Tau (*MAPT*) R | TGTCTTGGCTTTGGCATTCTC |  |
| β-catenin (*CTNNB1*) F | AGTCCTTCACGCAAGAGCAA |  |
| β-catenin (*CTNNB1*) R | TTAGTGGGATGAGCAGCGTC |  |
| IRS1 F | GGGAGATTCCAACACCAGCA |  |
| IRS1 R | ATCTTCGGCAGTTGCGGTAT |  |
| actin F | GGCTGTATTCCCCTCCATCG |  |
| actin R | CCAGTTGGTAACAATGCCATGT |  |
