# Supplementary material for: Danggui Shaoyao San Alleviates Early Cognitive Impairment in Alzheimer's Disease Mice Through IRS1/GSK3β/Wnt3a‐β‐Catenin Pathway
Source: Brain Behav. 2024 Sep 30;14(10):e70056. doi: 10.1002/brb3.70056 (PMC11440033; doi:10.1002/brb3.70056)
Supplement: Supplementary file 3 — Supporting Information [file BRB3-14-e70056-s001.docx]

**DSS Components（Positive ion）**

| **Number** | **m/z** | **Retention time (min)** | **MOLID** | **Compound** | **Class** | **Score** | **Formula** | **Mass Error (ppm)** |
| --- | --- | --- | --- | --- | --- | --- | --- | --- |
| 1 | 248.1128 | 0.71 | MOL005706 | Linamarin | Organooxygen compounds | 48.5 | C_10_H_17_NO_6_ | -0.3324 |
| 2 | 138.0549 | 0.73 | MOL003870 | Trigonelline | Unclassified | 52.8 | C_7_H_7_NO_2_ | -0.1420 |
| 3 | 158.0924 | 0.84 | MOL003800 | Citrulline | Carboxylic acids and derivatives | 52 | C_6_H_13_N_3_O_3_ | 0.1696 |
| 4 | 298.0898 | 0.86 | MOL002079 | Sarmentosin | Fatty Acyls | 50.9 | C_11_H_17_NO_7_ | 0.4300 |
| 5 | 197.0811 | 0.93 | MOL001858 | CANTHARIDIN | Unclassified | 47.5 | C_10_H_12_O_4_ | 1.3563 |
| 6 | 124.0395 | 1.06 | MOL000421 | Nicotinic acid | Pyridines and derivatives | 41.3 | C_6_H_5_NO_2_ | 1.8580 |
| 7 | 381.1160 | 4.07 | MOL000651 | Sweroside | Unclassified | 43.1 | C_16_H_22_O_9_ | 1.0427 |
| 8 | 595.1659 | 4.20 | MOL001414 | Safflor Yellow A | Cinnamic acids and derivatives | 56.4 | C_27_H_30_O_15_ | 0.2875 |
| 9 | 311.0762 | 4.20 | MOL002816 | bergenin | Unclassified | 46.5 | C_14_H_16_O_9_ | 0.2088 |
| 10 | 561.1947 | 4.38 | MOL009797 | Diosbulbinoside F | Prenol lipids | 50.5 | C_26_H_34_O_12_ | 0.8374 |
| 11 | 481.1702 | 4.46 | MOL001924 | Paeoniflorin | Unclassified | 58.5 | C_23_H_28_O_11_ | -0.5155 |
| 12 | 435.1281 | 4.92 | MOL009072 | Prunin | Polyketides | 58.1 | C_21_H_22_O_10_ | -1.2035 |
| 13 | 523.1800 | 5.03 | MOL005176 | Oleuropein | Prenol lipids | 47.4 | C_25_H_32_O_13_ | -1.8611 |
| 14 | 223.0598 | 5.08 | MOL005723 | Isofraxidin | Unclassified | 47.7 | C_11_H_10_O_5_ | -1.3432 |
| 15 | 481.1700 | 5.19 | MOL007004 | Albiflorin | Unclassified | 54.5 | C_23_H_28_O_11_ | -0.8863 |
| 16 | 163.0753 | 5.19 | MOL000249 | Methyl cinnamate | Cinnamic acids and derivatives | 48.8 | C_10_H_10_O_2_ | -0.1399 |
| 17 | 201.1639 | 5.46 | MOL004314 | Zerumbone | Prenol lipids | 43.7 | C_15_H_22_O | 0.5066 |
| 18 | 455.2062 | 5.51 | MOL003959 | Desoxylimonin | Steroids and steroid derivatives | 48.8 | C_26_H_30_O_7_ | -0.4675 |
| 19 | 135.1169 | 5.55 | MOL000130 | Camphor | Prenol lipids | 47.1 | C_10_H_16_O | 0.2606 |
| 20 | 165.0911 | 5.60 | MOL008936 | THYMOQUINONE | Organooxygen compounds | 49.4 | C_10_H_12_O_2_ | 0.4588 |
| 21 | 219.1744 | 5.67 | MOL000910 | Germacrone | Unclassified | 46.6 | C_15_H_22_O | 0.3986 |
| 22 | 207.0655 | 5.78 | MOL004678 | CITROPTEN | Coumarins and derivatives | 47.4 | C_11_H_10_O_4_ | 1.4888 |
| 23 | 217.1589 | 5.94 | MOL004249 | Procurcumenol | Prenol lipids | 47.7 | C_15_H_22_O_2_ | 0.8965 |
| 24 | 237.1849 | 6.43 | MOL004254 | Curcumol | Prenol lipids | 47.1 | C_15_H_24_O_2_ | 0.1439 |
| 25 | 149.0961 | 6.57 | MOL000475 | ANETHOLE | Phenol ethers | 44.7 | C_10_H_12_O | 0.2159 |
| 26 | 246.2428 | 6.75 | MOL001393 | Myristic acid | Fatty Acyls | 47.9 | C_14_H_28_O_2_ | 0.0744 |
| 27 | 471.3475 | 9.21 | MOL011157 | Ganoderiol B | Prenol lipids | 55 | C_30_H_46_O_4_ | 1.2708 |
| 28 | 135.0441 | 9.54 | MOL001332 | Mandelic acid | Benzene and substituted derivatives | 40.1 | C_8_H_8_O_3_ | 0.5911 |
| 29 | 495.3471 | 9.91 | MOL011219 | Ganoderic acid S | Prenol lipids | 42.5 | C_32_H_48_O_5_ | 0.4547 |
| 30 | 277.1799 | 10.31 | MOL005235 | Embelin | Unclassified | 54.7 | C_17_H_26_O_4_ | 0.2387 |
| 31 | 493.3290 | 10.37 | MOL004804 | Glycyrrhetinic acid | Prenol lipids | 48.3 | C_30_H_46_O_4_ | 0.3042 |
| 32 | 296.2583 | 14.75 | MOL007181 | Punicic acid | Fatty Acyls | 46.4 | C_18_H_30_O_2_ | -0.4104 |

Score: full score of 60 points, primary mass spectrometry accurate molecular weight matching (20 points), secondary mass spectrometry fragment matching (20 points), isotope distribution matching (20 points). The higher the score, the more accurate the qualitative; Mass error: the molecular weight deviation between m/z and the matched ion in the database. The smaller the absolute value, the more accurate it is.
