# Supplementary material for: Danggui Shaoyao San Alleviates Early Cognitive Impairment in Alzheimer's Disease Mice Through IRS1/GSK3β/Wnt3a‐β‐Catenin Pathway
Source: Brain Behav. 2024 Sep 30;14(10):e70056. doi: 10.1002/brb3.70056 (PMC11440033; doi:10.1002/brb3.70056)
Supplement: Supplementary file 4 — Supporting Information [file BRB3-14-e70056-s004.docx]

**DSS Components（Negative ion）**

| **Number** | **m/z** | **Retention time (min)** | **MOLID** | **Metabolites** | **Class** | **Score** | **Formula** | **Mass Error (ppm)** |
| --- | --- | --- | --- | --- | --- | --- | --- | --- |
| 1 | 711.2218 | 1.00 | MOL000732 | Stachyose | Organooxygen compounds | 55.4 | C_24_H_42_O_21_ | 2.5599 |
| 2 | 827.2693 | 1.04 | MOL000848 | Verbascose | Unclassified | 54.7 | C_30_H_52_O_26_ | 2.2420 |
| 3 | 191.0201 | 1.08 | MOL001456 | Citric acid | Carboxylic acids and derivatives | 56.9 | C_6_H_8_O_7_ | 1.8438 |
| 4 | 323.0992 | 1.11 | MOL000526 | Trehalose | Organooxygen compounds | 45.7 | C_12_H_22_O_11_ | 2.3941 |
| 5 | 421.1360 | 1.15 | MOL000655 | Loganic acid | Unclassified | 57.7 | C_16_H_24_O_10_ | 2.3311 |
| 6 | 115.0039 | 1.20 | MOL000602 | Maleic acid | Carboxylic acids and derivatives | 55.2 | C_4_H_4_O_4_ | 1.5232 |
| 7 | 117.0195 | 1.24 | MOL000346 | Succinic acid | Carboxylic acids and derivatives | 58 | C_4_H_6_O_4_ | 1.7463 |
| 8 | 125.0246 | 1.41 | MOL000484 | Phloroglucinol | Pteridines and derivatives | 55.4 | C_6_H_6_O_3_ | 1.7788 |
| 9 | 391.1234 | 3.76 | MOL004560 | Shanzhiside | Unclassified | 50.6 | C_16_H_24_O_11_ | -2.9451 |
| 10 | 399.0940 | 4.10 | MOL000216 | Scopolin | Unclassified | 48.1 | C_16_H_18_O_9_ | 2.1215 |
| 11 | 353.0884 | 4.26 | MOL001955 | trans-Chlorogenic acid | Organooxygen compounds | 48.1 | C_16_H_18_O_9_ | 1.7424 |
| 12 | 569.1885 | 4.54 | MOL005185 | Ligustroside | Organooxygen compounds | 45 | C_25_H_32_O_12_ | 1.6702 |
| 13 | 413.1458 | 4.80 | MOL011543 | Zizybeoside I | Organooxygen compounds | 49.4 | C_19_H_28_O_11_ | 1.1659 |
| 14 | 375.1667 | 4.86 | MOL001409 | Picrocrocin | Organooxygen compounds | 48.5 | C_16_H_26_O_7_ | 1.8848 |
| 15 | 193.0508 | 4.92 | MOL000360 | ferulic acid | Cinnamic acids and derivatives | 56.4 | C_10_H_10_O_4_ | 1.0277 |
| 16 | 223.0980 | 6.89 | MOL005900 | Dhelwangin | Organooxygen compounds | 49.2 | C_12_H_16_O_4_ | 1.9851 |
| 17 | 383.1508 | 7.90 | MOL001951 | Bergamottin | Prenol lipids | 48 | C_21_H_22_O_4_ | 2.3842 |
